# Targeting Energy Expenditure—Drugs for Obesity Treatment

**DOI:** 10.3390/ph14050435

**Published:** 2021-05-06

**Authors:** Carlos M. Jimenez-Munoz, Marta López, Fernando Albericio, Kamil Makowski

**Affiliations:** 1 School of Chemical Sciences and Engineering Yachay Tech University, San Miguel de Urcuquí 100119, Ecuador; carlos.jimenez@yachaytech.edu.ec (C.M.J.-M.); lo.al.marta@gmail.com (M.L.); 2 Department of Surfactants and Biotechnology, Institute for Advanced Chemistry of Catalonia (IQAC-CSIC), 08034 Barcelona, Spain; 3 CIBER-BBN, Networking Centre of Bioengineering, Biomaterials, and Nanomedicine, and Department of Organic Chemistry, University of Barcelona, 08028 Barcelona, Spain; 4 School of Chemistry and Physics, University of KwaZulu-Natal, Durban 4001, South Africa

**Keywords:** obesity, energy expenditure, thermogenesis, energy metabolism, FDA-approved, treatments under development, clinical trials, withdrawn treatments

## Abstract

Obesity and overweight are associated with lethal diseases. In this context, obese and overweight individuals infected by COVID-19 are at greater risk of dying. Obesity is treated by three main pharmaceutical approaches, namely suppressing appetite, reducing energy intake by impairing absorption, and increasing energy expenditure. Most compounds used for the latter were first envisaged for other medical uses. However, several candidates are now being developed explicitly for targeting obesity by increasing energy expenditure. This review analyzes the compounds that show anti-obesity activity exerted through the energy expenditure pathway. They are classified on the basis of their development status: FDA-approved, Withdrawn, Clinical Trials, and Under Development. The chemical nature, target, mechanisms of action, and description of the current stage of development are described for each one.

## 1. Introduction

Obesity can be defined as conditions characterized by excessive body fat. In 2016, there were about 650 million obese adults, and this number is increasing each year [1]. It is estimated that more people are currently dying as a consequence of overweight or obesity than underweight due to undernutrition [2]. Obesity commonly encompasses other health problems, particularly type 2 diabetes mellitus, coronary disorders, and an increased incidence of various forms of cancer, among other diseases. This susceptibility has been highlighted during the COVID-19 pandemic, where obesity has been associated with a higher risk of death among those infected [3]. Additionally, obesity is associated with unemployment and social disadvantages. To make a clear distinction from being just a risk factor, the World Obesity Federation and other organizations have recently declared obesity as a chronic progressive disease. Additional to health risks directly related to overweight, obesity presents different pathophysiology as strong homeostatic mechanisms promote further weight gain. Whereas healthy eating and exercise is undoubted the best way to prevent obesity, this can be not enough for individuals with already developed obesity [2]. 

Obesity has nowadays become a significant health and financial burden for developed and developing societies. 

Though overweight causes many medical disorders, obesity treatment is still not the first medical prescription of choice. However, modern society is under pressure to become fitter, which would not only save lives but also improve quality of life. Currently, exercise and diet followed by bariatric surgery are the gold standard treatment for obesity. While diet and exercise are the safest and healthiest approaches, it has been reported that they are only long-term effective in a small percentage of individuals [4]. On the other hand, bariatric surgery intervention come with some significant risk, so it is only used for extreme cases of obesity. 

**Figure 1:**
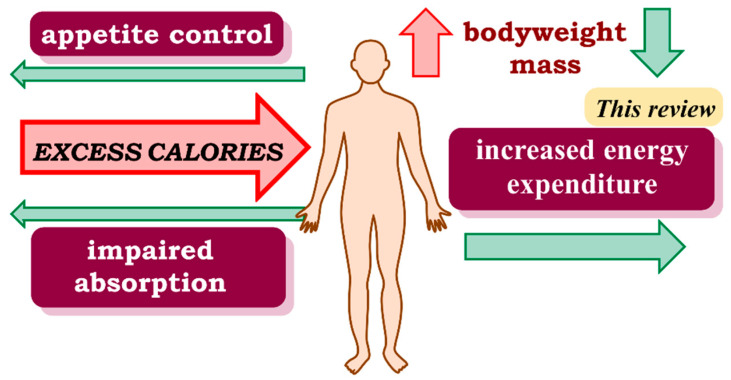
Excess intake of calories causes overweight and obesity. There are three main pharmacological strategies to achieve a caloric deficit. Two of them aim to prevent the storage of excess calories, first by acting on the central nervous system to cause an anorexic effect and regulate appetite, and second by inducing early excretion and thus preventing ingested food from becoming a useful source of calories. The third strategy, and the topic of this review, is the use of drugs that increase energy expenditure by causing the body to use calories already stored in the form of fat.

Therefore, in this context, there is an urgent and highly demanded need for the development of new drugs to treat obesity, either as an alternative or complement to diet, exercise, or bariatric surgery treatment. Three pharmacological approaches can be used to achieve a caloric deficiency that would lead to a loss of body weight: (i) decreasing energy intake by appetite suppression, (ii) reducing energy intake by impaired absorption, and (iii) increasing energy expenditure [5] (Figure 1). 

Principal concerns regarding pharmacological energy intake reduction strategy are the homeostatic mechanisms of the human body overcoming caloric restriction and long-term clinical efficacy. The energy expenditure strategy to fight obesity is crucial because it does not prevent the absorption of essential nutrients such as vitamins and minerals and it might be used alone or reinforce the calorific restriction strategies. Additionally, there are only a few available drugs that target energy expenditure, hence making their use an attractive and promising approach for obesity treatment. Identifying safe pharmaceutical products for this purpose is challenging because many compounds that have been studied do not show a high selectivity for their target, resulting in dangerous side effects. Nevertheless, a significant number of the drugs studied to date have reached clinical phases. 

There are several processes in the human body that are inefficient in terms of energy preservation, where energy is lost as heat and not used to drive another cellular process. In cases where energy is lost in a significant proportion, those processes are called uncoupled. An example of exothermic uncoupled processes involving muscle and adipose tissue is a futile cycle of the tricarboxylic acid (TCA). The heat releasing produces in TCA can be observed in burn injuries, cancer cachexia, exercise, and after lipolysis-increasing therapeutics. In this process, the hydrolysis of triglycerides liberate fatty acids (FAs) and heat, and then fatty acids converted to FA-acyl-CoA are esterified back to triglycerides to close the futile cycle [5]. 

The most representative example of uncoupled processes is thermogenic proton leak regulated by uncoupling protein 1 (UCP1). This protein is solely presented in brown adipose tissue (BAT), and the primary function of BAT is protection against cold, especially newborns. BAT tissue is gradually lost over age but can be still preserved in adults, and recent studies have suggested that it can be stimulated either by cold or drugs. UCP1 acts as a protonophore that alters the normal flow of protons between mitochondrial intermembrane space and the inner mitochondrial membrane in ATP production; this uncouples oxidative phosphorylation, which causes energy to convert from triglycerides into heat. UCP1 activation and thermogenesis, in general, are controlled by the sympathetic nervous system (SNS). The stimulation of an SNS through β-adrenoreceptors (β-ARs) increases cellular catabolism and triggers lipolysis in white adipose tissue. Liberated fatty acids in this process increase energy expenditure by (1) providing fuel for mitochondrial oxidation in brow adipocytes and (2) binding to and activating UCP1. Additionally, UCP1 gene transcription can be pharmacologically stimulated by the activation of peroxisome proliferator-activated receptor gamma coactivator 1-alpha (PGCα) and peroxisome proliferator-activated receptors α and γ (PPARα and PPAR γ). Furthermore, there is a class of compounds called sympathomimetic drugs that mimic the effects of the endogenous agonists of the SNS and, in several cases, present bodyweight loss activity through BAT activation mechanisms. 

In most tissues, thermogenesis is possible due to uncoupled mitochondrial processes as a consequence of cation leakage across concentrations gradients. Cation leakage produces increase ATP hydrolysis, and in this process, heat is released. For example, non-shivering thermogenesis in muscle is mediated by calcium cycling. Sarcolipin endoplasmic reticulum Ca^2+^-ATPase (SERCA) resides in the sarcoplasmic reticulum and BAT, and it actively pumps calcium cations during muscle relaxation. Sarcolipin treatment uncouples the hydrolysis of ATP from calcium cation transport, thus producing heat in a futile cycle of Ca^2+^ [6]. More recently, the creatine cycle as a new mechanism related to thermogenesis in beige adipocytes was reported [7]. Small molecules called protonophores or chemical uncouplers (such as 2,4-dinitrophenol, which is discussed later) similar to UPC1 (but not BAT limited) can uncouple oxidative phosphorylation, forcing the TCA cycle to run faster and produce heat. Interestingly, increase energy expenditure by protonophore treatment can be sustained without tolerance [5,8]. Modern approaches, either with molecular design or controlled-release drugs, aim to eliminate the dangerous secondary effect of chemical uncouplers.

Thermogenesis is regulated by the central nervous system, and, when stimulated, it can produce the overexpression of UCP1. An important example is the melanocortin 4 receptor (MC4-R) in the dorsomedial hypothalamus. After the injection of an MC4-R agonist, an increased BAT UCP-1 mRNA is observed [9]. Furthermore, hormonal regulation in the hypothalamus is important in thermogenesis. Besides appetite modulation, leptin increases sympathetic nerve activity and consequently increases energy expenditure in interscapular brown adipose tissue [10]. Leptin resistance can lead to obesity, but some drugs called leptin sensitizers can restore leptin sensitivity and produce a loss of body weight [11].

**Figure 2:**
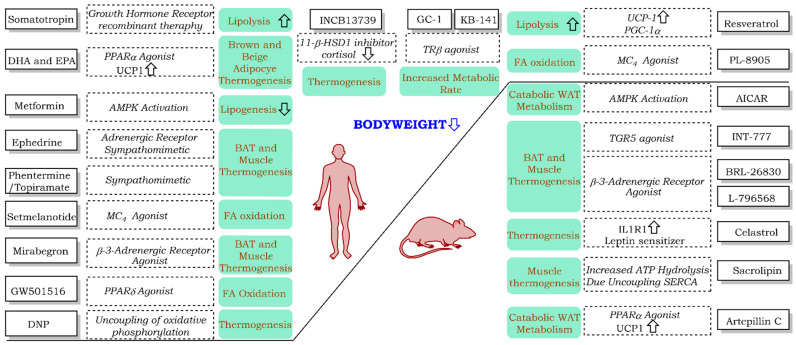
Drugs, their targets, and their metabolic outcomes focusing on energy expenditure effect.

This review provides a description of selected drugs that exert their action through the energy expenditure pathway, as well as their chemical structure and biological targets (Figure 2), classified on the basis of development status: FDA (US Food and Drug Administration)-approved, Withdrawn, Clinical Trials, and Treatments Under Development. Some of the drugs presented here are used to treat patients or have been studied in clinical trials for disorders different that obesity; however, each has shown an increased energy expenditure activity and is thus possible to use to decrease body weight in obese individuals. We believe that this analysis with an emphasis on drug chemical structures will be useful in developing medicinal chemistry projects focused on increased energy expenditure for obesity treatment. 

## 2. FDA -Approved and -Withdrawn Drugs 

**Figure 3:**
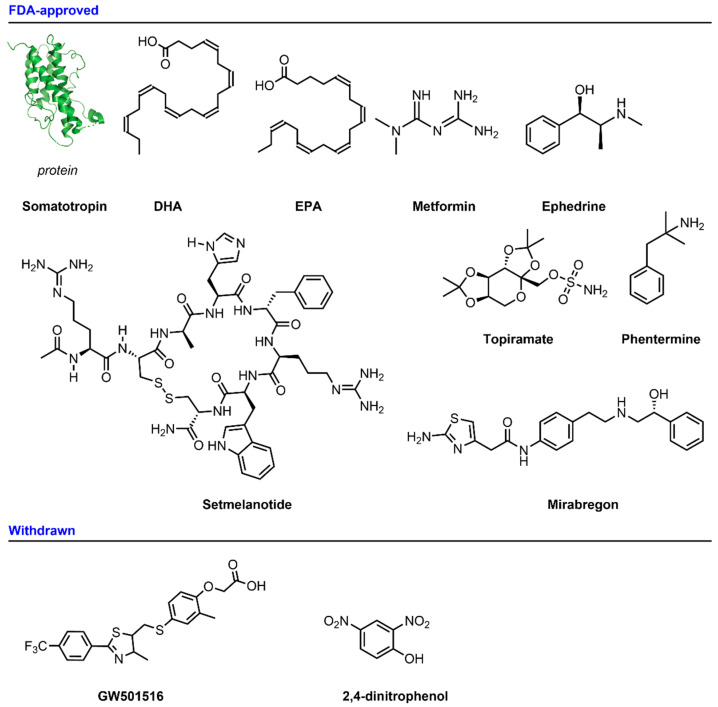
Chemical structures of drugs that increase energy expenditure that are approved by the FDA and withdrawn from the market.

Following are chemical structures of selected drugs that increase energy ex-penditure that are approved by the FDA and withdrawn from the market. (Figure 3)

### 2.1. FDA-Approved

#### 2.1.1. Somatotropin (HGH, Human Growth Hormone)

Somatotropin is a growth hormone of 191 amino acids that is synthesized in human and animal pituitary glands. This gland is responsible for the secretion of somatotropin, the main effect of which is the stimulation of growth during childhood and the maintenance of a healthy body by increasing the volume of muscles and the consumption of fat mass. Additionally, somatotropin is responsible for keeping the concentration of glucose in blood stable [12]. 

The spontaneous secretion of HGH is determined by variables such as age, fitness, and degree of adiposity. Normal secretion is mainly affected by body mass index (BMI), GH half-life, GH amplitude secretory episodes, and a pulsatile component of GH release [13]. 

Recent evidence has indicated that the decreased secretion of somatotropin in obese subjects is caused by increased somatostatinergic tone or/and peripheral mechanisms such as increased circulating levels of insulin and free fatty acids (FFAs). GH treatment results in a significant reduction in fat mass, increased fat-free mass, and increased resting energy expenditure in these subjects [14]. The deregulated production of this hormone is associated with deviations in body composition, unfavorable changes in cardiovascular function, and metabolic alterations in general [15]. The stimulated secretion of GH through pharmacological treatment oriented to the central neurotransmitter system has a favorable hormonal response that is capable of modifying the somatotropin response [13].

In a clinical assay developed in 2018 by Lian et al. [16], 43 obese children with relative GH deficiency were included in a study addressing treatment with recombinant human GH. Twenty-three of them received the treatment, while the remaining 20 individuals formed part of the control group. The treatment consisted of a weekly dose of 0.23–0.35 mg of recombinant human GH per kg of body weight, which was subcutaneously administered every night before bed. After six months of treatment, those children who received the treatment showed a reduction in body mass index standard deviation score and a positive impact on blood lipid profiles. Additionally, no significant effects or adverse effects were observed on insulin resistance or glucose homeostasis. 

A Phase IV clinical trial with somatotropin was recently proposed for obese individuals with COVID-19 to speed up recovery. However, the study was withdrawn before the enrolment of the first patient [17]. Somatotropin is marketed under various names, including Genotonorm, Humatrope, Norditropin, Nutropina, Omnitrope, and Zomacton, as well as Saizen for HGH replacement therapy. Most of these brands are used for clinical purposes only. Nevertheless, GH can be purchased relatively easily on the internet, where it is advertised as an anabolic supplement for bodybuilders and an enhancing agent for athletes [18]. 

#### 2.1.2. DHA and EPA

DHA and EPA (docosahexaenoic acid and eicosapentaenoic acid) are omega-3 fatty acids with a double bond between the third and fourth carbon, and they are included in the PUFA (polyunsaturated fatty acids) family. These compounds show the attenuation of high-fat diet-induced insulin resistance and potent anti-inflammatory effects, which inhibit macrophage infiltration into adipose tissue. DHA (doconexent) and EPA (icosapent) reduce insulin resistance [19].

n-3 PUFAs can affect weight loss through several mechanisms, such as by increasing fat oxidation and energy expenditure, inducing apoptosis in adipocytes, suppressing appetite, and inducing the gene expression of adipose tissue hormones [20]. Long-chain PUFAs ameliorate physiological conditions like hyperlipidemia, diabetes, cancer, inflammation, and neurodegenerative diseases. EPA can inhibit the synthesis of endogenous TAGs (triacylglycerols) and cholesterol. Long-chain n-3 PUFAs are natural ligands for several nuclear receptors that regulate gene expression, including the α isoform of peroxisome proliferator-activated receptors, related to energy homeostasis [21].

n-3 PUFAs trigger distinct transcriptional changes in the liver and skeletal muscle when supplemented. These transcriptional patterns are attenuated by DHA but amplified by EPA, which suggests that these fatty acids are nutritionally essential and have distinct biological effects [19,22]. n-3 PUFAs increase energy expenditure in mice by activating brown and beige adipocyte UCP1-mediated non-shivering thermogenesis [23].

Supplementation with DHA and EPA has a significant positive effect on biological parameters such as insulin (HOMA-IR), triglycerides, and LDL cholesterol [24]. 

Since their approval by the FDA, these two drugs are distributed by Glaxo Smith Kline (GSK) under the brand name Lovaza, by Astra Zeneca under Epanova and by Amarin Pharma under Vascepa, the latter containing only DHA. More recently, the FDA approved Omtryg, which is a formulation of DHA and EPA ethyl esters that is sold by Trygg Pharma AS, with a generic distributed by Teva Pharmaceuticals.

#### 2.1.3. Setmelanotide

Setmelanotide (RM-493) is a disulfide-containing cyclic octapeptide with an acetylated N-terminus and an amidated C-terminus. The peptidic chain contains two D-amino acids, Ala and Phe, the rest having an L-configuration [25]. Setmelanotide is a melanocortin 4 receptor (MC_4_ receptor) agonist that effectively treats obesity without the adverse cardiovascular effects caused by other MC_4_ receptor agonists. Located in the hypothalamus, the MC_4_ receptor is a crucial regulator of energy homeostasis that modulates food intake, increases energy expenditure, and causes weight loss when chronically activated [26]. 

Pro-opiomelanocortin (POMC) is the natural agonist ligand of the MC_4_ receptor. Setmelanotide is used as replacement therapy for POMC deficiency to treat obesity in genetically severe obesity disorders involving impaired POMC neuronal function [27,28].

In 2020, the FDA approved setmelanotide for chronic weight management in obese patients from six years onwards whose obesity is caused by POMC, LEPR, and proprotein convertase subtilisin/kexin type 1 deficiency (PCSK_1_). However, this drug causes some minor side effects, including injection site reactions, skin hyperpigmentation, headaches, and gastrointestinal side effects [29]. 

#### 2.1.4. Metformin

Metformin, a guanidine derivative, is a hydrophilic base and is absorbed predominately from the small intestine [30]. It is currently the first drug of choice for the treatment of type 2 diabetes, according to the guidelines of the American Diabetes Association and the European Association of the Study of Diabetes. 

Metformin causes a reduction in hepatic glucose production and diabetes-related complications. Its mechanism of action is not entirely understood; however, it has been shown to act via AMP-activated protein kinase (AMPK)-dependent and -independent mechanisms. 

Metformin also inhibits mitochondrial respiration and mitochondrial glycerophosphate dehydrogenase, as well as mechanisms that involve lysosomes. Furthermore, the activation of AMPK is closely linked to the action of metformin. AMPK activation by metformin induces the phosphorylation and inactivation of acetyl-CoA carboxylase, which regulates the enzyme responsible for synthesizing the malonyl-CoA precursor of the biosynthesis of fatty acids and an inhibitor of mitochondrial fatty acid oxidation [31]. 

Metformin regulates lipogenic gene expression. In human hepatoma cells (HepG2), metformin improves the acetyl-CoA carboxylase phosphorylation, thereby leading to a decrease in triacylglycerol levels (triglycerides). Furthermore, AMPK suppresses the expression of lipogenic genes such as FAS (fatty acid synthase) and ACC (acetyl-CoA carboxylase) by phosphorylating transcription factors. [31]. Furthermore, metformin induces the phosphorylation and repression of SCD1 (stearoyl-CoA desaturase 1). This enzyme is involved in the biosynthesis of monounsaturated fatty acids from saturated fatty acids. Metformin causes the considerable suppression of gluconeogenesis and improves insulin signaling in the liver [32]. 

More recent studies have shown that the impact of metformin is focused on caloric intake instead of energy expenditure. However, the mechanism by which this drug suppresses appetite is still being elucidated [33]. The role of metformin in obesity and overweight was recently reviewed [34]. The weight loss effect of the drug at the beginning of treatment was associated with energy expenditure then progressed to exerting an anorexic property. A recent study suggested that metformin exerts its action though a combination of both effects. Tokubuchi et al. reported that metformin reduces the volume of visceral fat, thereby improving energy metabolism, and they suggested that this effect could be due to forcing metabolism to switch to fat oxidation and the upregulation of adaptive thermogenesis, in addition to its anorexic effect [35]. 

Metformin is widely distributed worldwide by various companies under brand names such as Fortamet, Glucophage, Glumetza, Glycon, and Riomet. 

#### 2.1.5. Ephedrine

Ephedrine is an alkaloid similar to the recreational drug phenethylamine. In fact, it is a synthetic precursor containing an extra hydroxyl group and two stereocenters. Ephedrine is a hydroxy-substituted amphetamine. Interestingly, the amino-phenylethanol moiety of the ephedrine molecule is also present in BRL-26830 and L-796568 (as pyridyl instead of phenyl in this case). These three compounds have the β-3-adrenergic receptor as a biological target.

The mechanism of action behind treatments with sympathomimetic drugs in humans is still not fully understood. Sympathomimetic agents act via adrenergic receptors (ARs) and, as well as cold exposure, activate brow adipose tissue [36]. All ARs are expressed in adipose tissue and mediate most critical adipocyte functions related to energy substrate regulation and expenditure [37]. BAT is a sympathetically activated thermogenic organ. In rodents and humans, this organ turns excess energy into heat to maintain an energy balance, and it also acts as a thermoregulator for the body core in low temperatures [9].

The sympathomimetic action of ephedrine is the increased release of endogenous noradrenaline (NA) from sympathetic nerve terminals and the blockade of its reuptake. In humans, a decrease in ambient temperature may reduce body weight, potentially via increasing BAT activity. In mice, treatment with ephedrine increases BAT adaptative thermogenesis; in contrast, ephedrine treatment in humans leads to the downregulation of BAT activity [36]. 

Furthermore, caffeine and ephedrine have been used in combination to induce weight loss through increased energy expenditure and reduced appetite by binding to the β-adrenergic receptor (ephedrine) and adenosine receptor (caffeine) [38]. Of note, caffeine alone has only shown a weak anorexic effect [39]. Ephedrine is distributed under the brand names Akovaz, Corphedra, Emerphed, and Ephedrine Sulphate. 

#### 2.1.6. Phentermine/Topiramate

Phentermine is an atypical amphetamine analog substituted with an extra methyl group on the alpha carbon. Phentermine acts in the central nervous system by reducing the release of norepinephrine, dopamine, and serotonin. Due to its similarity to amphetamine, phentermine is a central nervous system stimulant, and there is an increased risk of addiction [40]. The FDA recently approved a combination treatment with phentermine and topiramate.

Topiramate is a hexose derivative in which the hydroxy group has been converted to the corresponding sulfamate ester. It is an antiepileptic that modulates voltage-activated sodium channels and calcium channels [41]. It mediates gamma-aminobutyric acid (GABA) receptor-mediated inhibitory currents, which are the most widely characterized inhibitory neurotransmitters in the brain, and it also mediates suppressing neuronal excitability. GABA is involved in the regulation of systemic metabolism [42]. Furthermore, topiramate is a potent inhibitor of the carbonic anhydrase enzyme and is thought to reduce appetite by altering taste [41]. 

The mechanism of action exerted by phentermine/topiramate is not fully understood. However, results suggest that phentermine suppresses food intake, while topiramate increases energy expenditure, thereby contributing to weight loss [43,44]. The phentermine/topiramate combination is one of the most potent weight loss treatments available, with a median weight loss of 10.2 kg at the maximum weekly dose of P 7.5 mg/T 46 mg over 52 weeks [40]. The phentermine/topiramate combination therapy was well-tolerated in Phase III clinical trials, the common main side effects being dry mouth, dizziness, constipation, insomnia, paresthesia, and dysgeusia [40]. 

The phentermine/topiramate combination therapy benefits from the actions of each drug, which have different effects on weight loss and allow the use of low doses of each one. phentermine/topiramate [45], which is presented in oral capsule format, is produced by Vivus Inc. and sold under the commercial name Qsymia.

#### 2.1.7. Mirabegron

Mirabegron is a monocarboxylic acid amide obtained from the condensation of a carboxy group with an anilino group. It is a β-3-adrenergic receptor agonist with good bioavailability, a high in vitro binding affinity, and specificity for the human β-3-adrenergic receptor. Mirabegron increases metabolic BAT activity and triggers WAT lipolysis [46]. 

The FDA approved mirabegron in 2012 for the treatment of overactive bladder syndrome (OAB) and later received authorization from agencies in Japan, the European Union, and Canada at a daily dose 50 mg. Regarding obesity treatment, a single dose of 200 mg of mirabegron induces an increase in whole-body energy expenditure and glucose uptake by BAT. However, this dose is higher than the approved therapeutic dose. High doses (200 mg) effectively activate BAT; however, the prolonged adrenergic stimulation of the cardiovascular system could potentially increase the risk of hypertension due to the β-3-adrenergic receptor losing selectivity. This dose indirectly activates a β-1-adrenergic receptor that is widely expressed in the cardiovascular system [47,48]. Mirabegron is distributed under the brand names Betmiga and Myrbetriq. 

### 2.2. Withdrawn

This section discusses those compounds that reached clinical trials or were available on the market but were later banned by the FDA.

#### 2.2.1. GW501516 

GW501516 is a member of the so-called small molecule class of drugs and it contains a 4-(trifluoromethyl)phenyl-4,5-dihydrothiazole moiety linked to a phenoxyacetic acid through a thioether.

GW501516 is an agonist of PPAR-δ that is involved in energy homeostasis. PPAR-δ is most abundant in muscle. In vivo trials showed that the GW501516 treatment of obese mice prevents massive fat accumulation; the effect was observed both in BAT and the liver. Muscle cells showed stimulated fatty acid oxidation [49]. GW501516 increases proglucagon gene expression and GLP-1 secretion, thereby suggesting a metabolic control of derangements in glucose homeostasis. In addition, it improves plasma lipid and protects against diet-induced obesity in mice [50].

The in vivo activation of PPAR-δ in adipose tissue leads to the upregulation of energy expenditure by fatty acid oxidation. Furthermore, by triggering the genes for the β-oxidation of fatty acids, GW501516 protects against obesity and fatty liver in mice fed a high-calorie diet. Additionally, it activates the heat-producing uncoupling enzymes in BAT and muscle [49]. GW501516 is involved in mitochondrial biogenesis. It also enhances the expression of FoxO1, a transcription factor involved in metabolic adaptation [51]. GW501516 was tested in Phase I and II clinical trials for metabolic disorders such as hypercholesterolemia and dyslipidemia, and it showed no significant side effects in humans [52,53].

While GW501516 shows promising anti-obesity effects under the controlled dosing of clinical trials, it is also associated with troubling side effects caused by non-specific interactions. This compound was used as a doping agent in sports; however, it was prohibited because of a non-specific interaction with PPAR-α instead of PPAR-δ, and it stimulated carcinogenesis in some animal models. Furthermore, this compound is linked to a decrease in bone density in ovariectomized rats, an effect shown by other PPAR-γ agonists, thereby indicating a non-specific interaction with the receptors [54].

However, despite the issues surrounding GW501516, it still shows fascinating anti-diabetic and anti-obesity activity. To ensure safety when using GW501516, modern drug delivery mechanisms could be tested to enhance targeted administration. In this regard, Wang et al. [55] used polymer-encapsulated doses of GW501516 and achieved the controlled release of the drug.

GW501516 is not available legally in the pharmaceutical market; however, it can be acquired on the black market under the brand names Cardarine and Endurobol. GW501516 was prohibited by the U.S. Anti-Doping Agency [56] and the World Anti-Doping Agency (WA-DA) as metabolic modulator substance [57].

#### 2.2.2. 2.4-Dinitrophenol

2,4-dinitrophenol (DNP) is a product of 2,4-dinitrochlorobenzene and explosive when not in solution. In the early 1930s, it was widely available without prescription after the discovery that its consumption led to weight loss. After a few years, it became clear that DNP ingestion was associated with lethal side effects such as hyperthermia, tachycardia, and decreased blood pressure. In 1938, the Federal Food, Drug, and Cosmetic Act announced that DNP was “extremely dangerous and not fit for human consumption” [58].

Nevertheless, it is still possible to buy DNP on the internet as a “fat burner,” which is popular among the bodybuilding community. A study by Petróczi et al. provided an analysis of ninety-eight pre-workout and weight-loss supplements containing DNP acquired from internet [59]. However, fatalities are commonly reported after consuming this compound. The toxicological effects of DNP, together with fatalities since 1916, were recently reviewed. The acute (short-term) effects of oral DNP in humans include nausea, vomiting, sweating, dizziness, headaches, and weight loss. Chronic (long-term) oral exposure to DNP in humans has results in ocular lesions, skin lesions, and a negative impact on the bone marrow, central nervous system, and cardiovascular system [60].

The increased energy expenditure induced by DNP is caused by an increase in body temperature. This effect is due to the chemical uncoupling of the oxidative phosphorylation process and the lowering of coupling efficiency, which means a decreased percentage of oxygen used to produce ATP. DNP is a protonophore and can alter the ATP/ADP equilibrium between the inner and outer membrane of matrix mitochondria by causing them to release one of their protons and increasing the concentration of H^+^ in the matrix without ATP [58]. This process alters the metabolic cycle and lowers coupling efficiency, and under these conditions, fat is burned and heat is produced. 

In a study in which four volunteers were placed on various diets (balanced, high carbohydrate, high fat, or increased protein) and given an average dose of 3.53 mg/kg/day of DNP for 7–16 days, the average weight loss at the end of the treatment was approximately 2 pounds (0.92 kg). Interestingly, the type of diet did not appear to influence the degree of weight loss [61]. More recently, in dose-ranging experiments in mice, the authors found a balanced dose (about 89 mg/kg/day) where the compound did not produce side effects like reduced water and food intake but did cause a loss of fat mass, reduced hepatic steatosis, and improved glucose tolerance [62]. 

Many efforts have been made to find a DNP derivative and develop new mitochondrial uncouplers with less toxicity [63]. In this regard, the most promising compound in the context of obesity treatment is an orally bioavailable CZ5 protonophoric uncoupler with favorable pharmacokinetics demonstrated in vivo. Single doses of 500 mg/kg/day of this drug in mice did not cause toxicity. Prolonged administration at 10 mg/kg per day to obese mice led to a reduction in fat mass and generally improved metabolic parameters [64]. New anilino pyrazines were found as a potential mitochondrial uncoupler; however, there are still no biological activity data available [65]. These findings suggest that chemical uncouplers may have interesting applications in the context of treating obesity. 

## 3. Clinical Trials 

**Figure 4:**
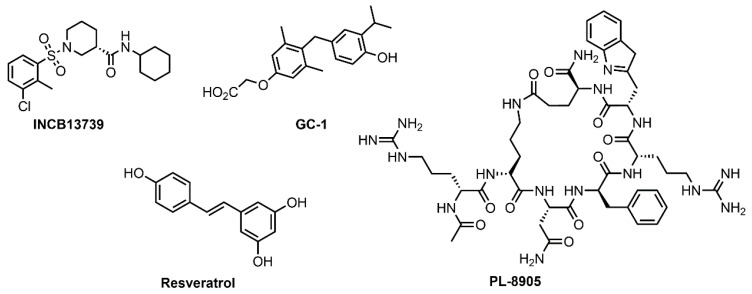
Drugs that increase energy expenditure in clinical trials.

Following are Drugs that increase energy expenditure in clinical trials (Figure 4)

### 3.1. INCB13739 (Oxophenylarsine)

This drug is an 11-β-hydroxysteroid dehydrogenase type 1 (11-β-HSD1) inhibitor. The inhibition of this enzyme became a therapeutic target for treating metabolic disorders, including type 2 diabetes mellitus and obesity [66]. The principal characteristic of this compound is its high selectivity over other dehydrogenases, glucocorticoid, and mineral corticoid receptors [67].

11-β-HSD1—an NADPH-dependent enzyme expressed in the liver, adipose tissue, vasculature, and brain—increases intracellular cortisol levels [68]. The primary effect of 11-β-HSD1 is the conversion of inactive cortisone to cortisol hormone. Cortisol increases sugar levels in the blood and enhances the metabolism of lipids, proteins, and carbohydrates [66]. 

According to Henry et al. [69], high cortisol responder sheep show increased food intake and reduced energy expenditure; thus, cortisol responsiveness can modify energy expenditure by producing thermogenesis. The importance of 11-β-HSD1 activity was recognized in glycemic regulation and as a cardiometabolic risk [70]. The benefits of 11-β-HSD1 are higher in patients with a BMI superior to 30 kg/m^2^. This observation thus indicates the relevance of this receptor as an anti-obesity target [70].

In a study with type 2 diabetes, patients where ongoing metformin (1.5 g daily dose) was carried out, INCB13739 was added over 12 weeks with daily dose between 5 and 200 mg. Those patients treated with the highest dose (200 mg) of INCB13739 presented reduced sugar levels in blood, fasting plasma glucose, and insulin resistance. Additionally, patients who completed the trial lost between 0.6 and 1.1 kg of body weight, depending on the daily dose. Furthermore, a decrease in total cholesterol was observed, falling by 7 mg/dL in the group receiving 200 mg of INCB13739. Moreover, coadministration with metformin was well-tolerated and improved glycemic response compared to monotreatment with metformin [66].

### 3.2. GC-1 (Sobetirome or QRX-431) 

GC-1 is a modified phenoxyacetic acid and selective thyroid hormone receptor modulator that binds to and selectively activates the β-isoform of the thyroid hormone receptor (TR-β). This receptor activation causes thyroid hormone resistance and regulates serum cholesterol [71,72]. TR-β receptors regulate body weight, adiposity, cholesterol levels, and possibly increase the metabolic rate [73]. Modulation of this receptor is associated with decrease in body weight and anti-diabetogenic properties [71,74].

The alpha-isoform (TR-α) of the thyroid hormone, which also accumulates in the liver, is responsible for thyrotoxic effects on the heart, muscles, and bones [71,72]. Additionally, TR-α receptors regulate the heart rate [75]. 

GC-1, also called QRX-431 and sobetirome, decreases the plasma levels of triglycerides and lipoproteins, as well as inducing a loss of fat. It can also be used to stimulate hepatic pathways that reduce cholesterol [71]. In preclinical animal studies and Phase I human clinical trials with GC-1, cardiovascular disorders remain a significant side effect. An excess thyroid hormone level leads to an elevated heart rate, arrhythmias, bone and muscle catabolism, mood disturbances, and reductions in serum cholesterol and body fat [72]. GC-1 has all the beneficial metabolic properties of the active thyroid hormone form (3,5,3′-triiodo-l-thyronine (T3)). Interestingly, this compound reached clinical trials and was studied for efficacy in a rare nervous disease called X-linked adrenoleukodystrophy but was withdrawn [76].

### 3.3. Resveratrol

Resveratrol (3,5,4′-trihydroxy-*trans*-stilbene) is a natural product that can be extracted from several plants. It is a non-flavonoid polyphenol that is able to transfer hydrogen atoms to interrupt oxidative cascades in reactive species. This molecule can be found in two isoforms, namely *trans*- and *cis*-resveratrol. The *trans* isomer is the most stable steric form and consequently the preferred form in nature [77]. Smaller amounts of resveratrol can be found in peanuts, grapes, red wine, and mulberries [78].

The daily ingestion of 200–400 mg/kg body weight/day of resveratrol in four-to-eight-week-old male mice for 9–15 weeks resulted in a reduction in body fat and a lower weight of adipose depots. These results demonstrated an unequivocal anti-obesity effect. A histomorphological analysis of epididymal white adipose tissue (WAT) showed smaller adipocytes upon resveratrol treatment [77]. The resveratrol results in mice on high-fat diets were not due to decreased food intake but to a significantly increased basal energy expenditure and an enhanced adaptive thermogenesis capacity. A morphometric analysis of BAT revealed the presence of larger mitochondrial structures in resveratrol-treated mice [77].

The thermogenic mechanism of resveratrol is based on an increase of UCP1 expression and the gene expression of PPAR-γ with PGC-1α [77]. Resveratrol was identified as a small-molecule activator of sirtuin 1 (SIRT1), which requires nicotinamide adenine dinucleotide (NAD^+^) to perform deacetylation. This dependence links the activity of SIRT1 and cellular energy levels (NAD^+^). However, it is not clear whether the activation effect of resveratrol is due to targeting SIRT1 or the activation of AMPK [78,79]. 

Despite promising results in animal models, in which resveratrol has been shown to cause energy expenditure and stimulate weight loss, a long-term study of this compound in humans revealed that it does not affect body weight or body composition after a six-month treatment [80]. 

### 3.4. PL-8905

For obesity treatment, another agonist of MC4r, PL-8905 (a cyclic peptide), also shows high selectivity for this receptor over MC1r, presents minimal side effects on blood pressure, and has significant chemical and metabolic stability [81]. The company Palatin, inventor of the series of cyclic peptide MC4r agonists, including PL-8905, announced the clinical trials of this compound. However, to the best of our knowledge, there has been no official clinical study register of this compound to date. The decrease of food intake and energy expenditure via MC4r binding has emerged as a promising strategy for obesity treatment. 

## 4. Treatments under Development

**Figure 5:**
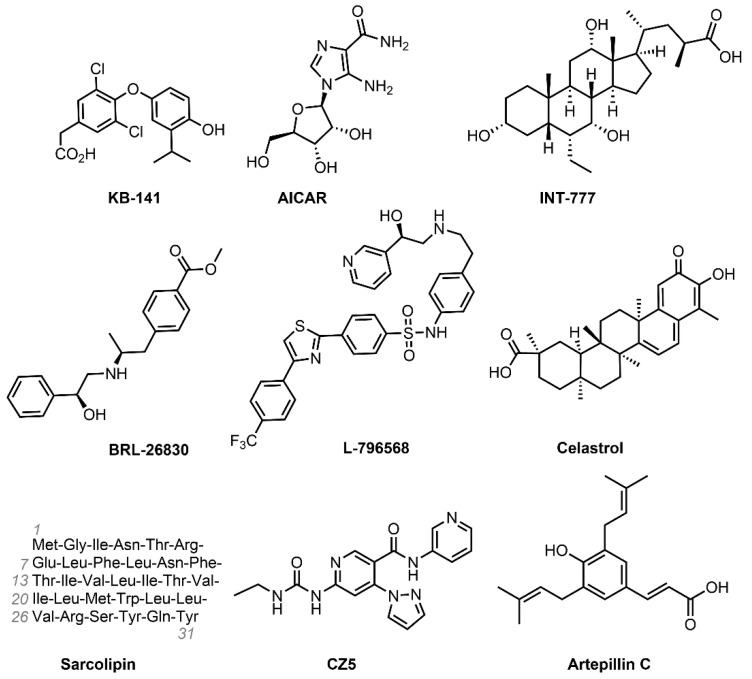
Drugs under development that increase energy expenditure.

Following are chemical structure of Drugs that are under development (Figure 5)

### 4.1. KB-141 (IH-5)

Like GC-1, KB-141 is a thyroid hormone receptor modulator, and it shares the same 4-hydroxy-3-isopropylfenil moiety. It shows a 14-fold greater affinity to TR-β than TR-α. Furthermore, it can reduce cholesterol and lipoprotein levels, and, in general, it can decrease body weight [82]. It has been described to have minimum cardiac side effects. Despite outperforming GC-1, it was not tested in clinical trials, probably for safety reasons. Cardiac side effects are a significant drawback in the development of TR agonists. To achieve an effective and safe treatment targeting the TR, ligands must show high affinity and extremely high selectivity for the β-isoform to avoid these side effects [83,84].

### 4.2. AICAR (Acadesine)

5-aminoimidazole-4-carboxamide ribonucleotide (AICAR) is a nucleotide that acts as an indicator of low energy states. It was first developed to keep blood flow during heart surgery and prevent ischemia, but more recently, it has also shown potential against diabetes. AICAR has a chemical structure analogous to that of adenosine monophosphate (AMP). The drug can activate AMPK, responsible for regulating homeostatic processes inside the cell. PPAR-γ is a ligand-activated transcription factor in a nuclear receptor. This receptor is present in almost all tissues in the body, and it is highly expressed in adipose tissue. Its primary actions are to regulate adipogenesis, energy metabolism, and cell proliferation. It was proposed as a target for obesity treatment [85]. In general, AMPK activation induces the inhibition of energy-consuming processes and switches on catabolic ATP-producing sites [86]. Gaidhu et al. showed that AMPK activation induced by AICAR increases the mRNA expression of PPAR-γ and its co-activator PGC-1α, resulting in a reduction of food intake, an increase in energy expenditure, and a decrease in visceral and subcutaneous adipose tissue [87].

Other studies have shown that AICAR improves the expression of β-catenin and its nuclear accumulation and also reduces the expression of the genes in charge of adipogenesis, namely PPAR-γ, the CCAAT/enhancer-binding protein (C/EPB)α, the fatty acid-binding protein (FABP)4, and lipoprotein lipase (LPL) [88]. Additionally, AICAR treatment inhibits intracellular processes like triglyceride accumulation [88].

### 4.3. INT-777

INT-777 is a semi-synthetic bile acid with a steroid structure. It is one of the most studied TGR5 (Takeda G-protein-coupled receptor 5) agonists. TGR5 is a bile acid-activated receptor (BAR) that, after inhibition, leads to improved glucose tolerance and a substantial reduction in reduce visceral adiposity [89]. TGR5 is a member of the rhodopsin-like family of transmembrane G protein-coupled receptors (GPCRs), which are expressed in multiple tissues, especially the colon and liver. Bile acid-stimulated TGR5 signaling controls type 2 deiodinase expression, which, in turn, regulates energy expenditure [90]. INT-777 also induces the renal expression of master regulators of mitochondrial biogenesis, oxidative stress inhibitors, and inducers of fatty acid β-oxidation [91]. 

A gene expression profiling study of BAT showed that the activation of TGR5 signaling pathways induces an increase in the expression of mitochondrial genes directly related to energy expenditure. Consequently, INT-777 activity triggers thermogenesis in BAT and the muscle, thereby causing energy expenditure [92].

INT-777 also controls DGAT1, an enzyme that is fundamental for triglyceride synthesis that increases PPAR-α, a critical nuclear receptor for fatty acid oxidation. Furthermore, INT-777 increases UCP2 and increases carnitine palmitoyltransferase Ib, which is responsible for transporting fatty acids from the cytosol into mitochondria and is the key enzyme involved in the β-oxidation of fatty acids. Thus, INT-777 causes decreased renal neutral lipid accumulation. All these properties endorse the capacity of this compound to reduce plasma triglycerides [91,93]. 

Finally, mice treated with INT-777 show reduced body weight, improved glucose tolerance, and improved insulin sensitivity. Furthermore, this compound increases PC1 expression and stimulates GLP-1 release. Additionally, INT-777 boosts pancreatic β-cell proliferation and insulin synthesis [94]. INT-777 is commercially available from several chemical suppliers for research purposes only. 

### 4.4. BRL-26830 and L-796568

These two compounds are β-3-adrenergic receptor agonists. The β-3-adrenergic receptor is the predominant adrenergic receptor in brown adipocytes, and its modulation triggers UCP1 expression, thereby increasing oxygen consumption and energy expenditure in BAT. The β-3-adrenergic receptor is widely known as a therapeutic target to treat obesity and metabolic diseases. The relevance of this receptor is due to the fact that it is present predominantly in adipose tissue but scarce in most other tissues. This distribution is a tremendous advantage for the selective targeting of obesity. An agonist of this receptor activates thermogenesis in adipose tissue, stimulating lipid oxidation, stimulating glucose consumption, and producing heat without causing cardiovascular side effects. It also activates BAT, thereby increasing energy expenditure [95]. Of note, recent findings suggest that in human BAT, UCP1 expression may be primarily regulated by β-1-adrenergic receptor and not like in mouse β-3-adrenergic receptor, but this is still under consideration [96].

BRL-26830 is a methyl ester of a benzoic acid derivative. In vivo studies in animals have shown that this compound acts through agonistic binding to a β-3-adrenergic receptor, causing a dose-dependent loss of body weight in obese rats and mice but not in lean counterparts. BRL-26830 increases energy expenditure and has no effect on caloric intake, thereby preserving lean mass but reducing overweight. Furthermore, this compound shows an increased BAT utilization. BRL-26830 in humans gave unimpressive results on overall body weight. However, most of these studies were reported 40 years ago, suggesting that novel studies are required to evaluate its activity as a β-3-adrenergic receptor agonist [97].

L-796568 contains the *N*-phenylbenzenesulfonamide moiety as a core with two substituents. One of the substituents, similar to GW501516, is a dihydrothiazol moiety and fluorinated methyl in the methylphenyl group, and another substituent is alkanolamine with a pyridine heterocycle. 

L-796568 is recognized as a full agonist of the human β-3-adrenergic receptor, with a high potency (Ec50 = 3.6 nmol/L) and a high specificity, thus causing fat mass changes [98]. However, in a study involving obese non-diabetic young men, it was not found to trigger a chronic thermogenic effect [99]. Nevertheless, in the same year as that study, it was revealed that energy expenditure and lipolysis were increased in overweight non-smoking male volunteers between 18 and 45 years of age at four hours post-administration of a 1000 mg dose [98]. Given that L-796568 showed high potency and specificity, further studies should be conducted to clarify this discordance in order to help with the development of obesity treatments with new analogs. 

### 4.5. Celastrol

Celastrol is a natural pentacyclic triterpene product extracted from *Tripterygium wilfordi*, and it has a long history of use for rheumatoid arthritis in traditional Chinese medicine. Celastrol reportedly has anti-cancer effects both in vitro and in vivo [100]. Celastrol is a leptin sensitizer and has been reported to have anti-obesity effects via adipogenesis inhibition, increased metabolic energy expenditure, and mitochondrial gene expression in mice fed a high-fat diet [101].

Leptin is a hormone produced by adipocytes, which regulate the amount of stored fat. If fat in an organism reaches high levels, leptin induces a reduction in appetite, an increase in body temperature, and an increment in energy expenditure [102]. Endoplasmic reticulum (ER) stress in the brain leads to leptin resistance and consequently obesity [100]. Leptin resistance can be reversed using “leptin sensitizers.” These compounds are able restore leptin sensitivity by targeting distinct neuroendocrine systems important in leptin signaling pathology, including ER stress and hypothalamic inflammation [11,103]. 

Celastrol causes a reduction in appetite and dramatic weight loss in hyper leptinemic diet-induced obese mice but not in leptin-deficient and leptin receptor-deficient mouse models [100]. In addition, celastrol increases the expression of heat shock transcription factor 1 (HSF1) and PGC-1α in the skeletal muscle and adipocytes of diet-induced obese mice, therefore promoting thermogenesis and the remodeling of WAT [101].

### 4.6. Sarcolipin

Sarcolipin (SLN) is a 31-amino acid mini-protein expressed only in striated and cardiac muscle. SLN regulates sarcoplasmic/endoplasmic reticulum Ca^2+^ transport ATPase (SERCA), which is involved in heat production by muscles. SLN creates energy demand via futile SERCA activity, increases ATP hydrolysis, and, consequently, induces thermogenesis [104,105].

In vivo assays in mice have shown that SLN overexpression causes resistance to high-fat diet-induced obesity and protects against lipotoxicity in muscle, thereby suggesting that higher SLN levels lead to enhanced energy expenditure through increased mitochondrial biogenesis [93]. Additionally, the overexpression of SLN in fast- and slow-twitch fibers leads to increased energy expenditure and resistance to high-fat diet-induced obesity. Increased SLN expression can lead to a higher energy cost for muscle, leading to less fat deposition. The overexpression of SLN leads to higher oxygen and caloric consumption, as well as greater mitochondrial biogenesis, in skeletal muscles. These observations can be attributed to increased oxidative metabolism. On the other hand, Maurya et al. [106] showed that mice lacking SLN are prone to diet-induced obesity. Though SLN is not a typical small-molecule therapeutic, it has excellent potential for the pharmacological treatment of obesity.

### 4.7. Artepillin C

Artepillin C (ArtC) is a product of the honeybee (*Apis mellifera*). The natural healing wonder, Brazilian green propolis, is mainly made from *Baccharis dracunculifolia* DC (Asteraceae) extracts and has been found to contain ArtC, among other compounds [107]. ArtC is a phenolic core trisubstituted with an α–β unsaturated carboxylic acid.

The extract itself triggers an increased expression of leptin protein in adipose tissue in mice and can improve metabolic states in obese individuals [107]. Moreover, the oral administration of ArtC (10 mg/kg) in mice leads to the significant formation and regulation of brown-like adipocytes and protein in inguinal WAT (iWAT) [108]. A more recent study showed that ArtC promotes thermogenesis in vivo, acting as a PPAR-γ agonist and stabilizing the PRD1-BF-1-RIZ1 homologous domain-containing protein, which is required for the development of beige adipocytes. Beige adipocytes and classical brown adipocytes release excess energy through UCP1 as heat [109].

Table 1 summaries all drugs revised herein.

## 5. Discussion

Regarding obesity and energy expenditure, many receptors and mechanisms have been identified to play a role in obesity treatment; however, by 2020, only a few treatments had been approved by the FDA. Current drug development to treat obesity is an intriguing field that is in constant evolution. Furthermore, it is crucial to consider that these drugs respond to a medical need and often to a social requirement. In this regard, some drugs that were studied and failed to gain approval may be available on the black market and could therefore significantly impair health due to the side effects they cause.

FDA-approved treatments include somatotropin, DHA and EPA, setmelanotide, metformin, ephedrine, phentermine/topiramate, and mirabegron. All of those compounds increase energy expenditure, but most of them are approved for disorders other than obesity such as insulin resistance, type 2 diabetes, hypertension, and overactive bladder. In some cases, these drugs are limited to clinical use, but they can also be acquired under prescription. On the other hand, DHA and EPA (fish-derived omega-3 fatty acids) are approved as dietary supplements and can be acquire without the prescription.

Cardarine and 2,4-dinitrophenol (DNP) show potent activity against obesity by increasing energy expenditure. However, these drugs have dangerous side effects that impede the development of an effective treatment derived from them. Remarkably, despite the demonstrated lethal consequences of using these drugs, they can be obtained on the black market as fat-burners and doping agents. Mitochondrial uncouplers such as DNP are currently gaining importance, mainly as derivatives, in the search for an effective treatment for obesity that does not cause side effects. 

A group of drugs, namely INCB13739, GC-1, resveratrol, and PL-8905, that have showed positive results against obesity reached clinical trials. Most of the studies for obesity treatment are ongoing or have concluded; however, in the case of GC-1, despite showing energy expenditure activity for obesity, clinical trials were carried out not only for the treatment of this condition but also of X-Linked adrenoleukodystrophy. Most of these drugs have been tested in Phase I or Phase II. Resveratrol, a natural product present in food, is under study in many clinical trials. The conditions under investigation with resveratrol include skin condition and inflammatory related factors, Friedreich ataxia, congestive heart failure, cognitive change, diabetes mellitus type 2, obesity, and COVID-19. Some of the trials consider to use this compound as a dietary supplementation.

The most relevant drugs under development are AICAR, INT-777, BRL-26830, L796568, celastrol, sarcolipin, and artepillin C. These drugs have not yet reached clinical trials, but preclinical studies have shown propitious results for the further investigation as anti-obesity drugs. 

## 6. Conclusions

Obesity and overweight pose some of the most dangerous health problems faced by modern society. Obesity is one of the ten principal causes of death worldwide (diabetes mellitus), and the number of individuals with obesity is growing each year. The impact of overweight and obesity has increased due to socio-cultural factors such as sedentarism and fast food with a low nutritional content. Furthermore, the high consumption of sugars and fats exacerbates the prevalence of these two conditions. 

Obesity is currently treated via three main approaches: suppressing appetite, reducing energy intake by impaired absorption, and increasing energy expenditure. Regarding the latter, a significant number of the drugs studied for anti-obesity treatment have reached Phase II or even Phase III clinical trials—not only for obesity but also for other diseases. 

The multiple risk factors caused by obesity call for the development of pharmacological treatments to avoid fatal consequences. In this regard, a group of treatments has been clinically tested and approved by the FDA; however, due to possibly irresponsible use, FDA-approved drugs are limited to clinical practice, and they are also only marketed under prescription in some cases. This strict control has led to the illegal distribution of anti-obesity drugs on the black market, especially Cardarine and DNP.

The world of fitness is another socio-cultural sector that plays a relevant role in the use of anti-obesity drugs. The illegal distribution of these drugs can be attributed to their use as doping agents and fat-burners, despite the counter-indications published in the literature. Self-medication and the lack of control regarding the availability of these drugs on the market can lead to acute side effects and even mortality.

We envisage that the new generation of anti-obesity drugs will be developed in medicinal programs that explicitly focus on the cause of obesity, and they will therefore have a minimum of secondary effects. 

## Figures and Tables

**Table 1:** Drugs Targeting Energy Expenditure for Antiobesity.

Compound	Target	Comment	Status	References
FDA-approved				
Somatotropin	Growth hormone receptor	Somatotropin reduces body mass in children without apparent side effects.	Phase IV (complete) for HGH deficiency, dwarfism, and obesity.	[12,13,14,15,16,17]
DHA and EPA	PPAR-α	n-3 PUFAs have shown weight loss mechanisms, such as increasing fat oxidation and energy expenditure, suppressing appetite and inducing apoptosis in adipocytes.	Phase IV (Completed) for obesity, insulin resistance, and as a dietary supplement.	[19,20,21,22,23]
Setmelanotide	Melanocortin 4 receptor (MC_4_r)	Setmelanotide (RM-493) is an MC_4_ receptor agonist approved by the FDA in 2020 to treat obesity. It does not have the adverse cardiovascular adverse effects shown by other MC_4_ receptor agonists.	FDA-approved and market distributed for leptin receptor deficiency, obesity, and POMC deficiency.	[25,26,27,28,81]
Metformin	AMP protein kinase	Metformin is currently the drug of first choice for the treatment of type 2 diabetes, following the guidelines of the American Diabetes Association and European Association of the Study of Diabetes.	FDA-approved for type 2 diabetes.	[30,31,32,33,34]
Ephedrine	Adrenergic receptors (ARs)	Ephedrine is defined as a sympathomimetic agent that replicates the adaptative thermogenic effects of chronic cold exposure in rodents; however, the treatment mechanism with sympathomimetic drugs in humans is still unknown.	FDA-approved for hypotension.	[36,37,38]
Phentermine/Topiramate	GABA receptor Carbonic anhydrase	Phentermine/topiramate: this combination of two drugs presents a very potent weight loss activity. After 52 weeks of treatment, an impressive median weight loss of 10.2 kg was produced at the maximum dose.	FDA-approved for chronic weight management.	[40,41,42,43,45]
Mirabegron	β-3-adrenergic receptor	The FDA approved mirabegron in 2012 for the treatment of overactive bladder syndrome (OAB). Approval at a daily dose of 50 mg was later given by other agencies from different regions, including Japan, the European Union, and Canada.	FDA-approved for overactive bladder.	[46,47,48]
Withdrawn				
GW501516 (Cardarine)	PPAR-δ (Peroxisome proliferator-activated receptor)	GW501516 is an anti-diabetic and anti-obesity treatment; however, because of the low specificity demonstrated in animal models, the oral administration of this compound is not considered a safe treatment.	Withdrawn from the market.	[49,50,51,52,54,55]
2,4-Dinitrophenol	Mitochondrial oxidative phosphorylation	DNP was prohibited by the FDA in 1938. The modern chemical uncoupler CZ5 has shown no toxic effects in vivo.	Withdrawn from the market.	[58,60,62,63,64,65]
Clinical Trials				
INCB13739	11-β-hydroxysteroid dehydrogenase type 1 (11-β-HSD1) inhibitor	Improves positive metabolic response in obese males with 2 diabetes mellitus, with a 12-week treatment leading to a body weight reduction of between 0.6 and 1.1 kg.	Phase II for insulin resistance, obesity and type 2 diabetes.	[67,68,69,70]
GC1	β-thyroid hormone receptor	GC-1 possesses all the beneficial metabolic properties of the active form of the thyroid hormone; however, preclinical animal studies and Phase I human clinical trials were withdrawn.	Phases I and II (for X-Linked adrenoleukodystrophy) but withdrawn.	[60,61,65,70]
Resveratrol	AMPK–SIRT1–PGC-1α axis	Resveratrol is a non-flavonoid polyphenol that has given promising results in animal models, producing energy expenditure and stimulating weight loss. However, a long-term study in humans showed that it does not affect body weight or body composition after 6 months of treatment.	Many different clinical trials concluded and ongoing, including Phase II.	[77,78,79,80]
PL-8905	Melanocortin 4 receptor (MC_4_r)	PL-8905 is a macrocyclic peptide that shows a high selectivity for the MC_4_ receptor. In preclinical obesity, models have shown weight loss and glucose regulation. This compound has minimal side effects.	Clinical trials announced.	[26,81]
Under Development				
KB-141	β-thyroid hormone receptor	KB-141 increases the metabolic rate and reduces the levels of plasma cholesterol without apparent cardiac side effects such as tachycardia.	Pre-clinical.	[73,74,83,84]
AICAR	AMP-activated protein kinase	AICAR can activate AMP-activated protein kinase (AMPK), which induces the inhibition of energy-consuming processes in numerous ways, switching on catabolic ATP-producing sites.	Pre-clinical.	[85,86,87,88]
INT777	TGR5 (Takeda G-protein-coupled receptor 5)	INT-777 activity triggers thermogenesis in brown adipose tissue (BAT) and muscle, causing energy expenditure.	Pre-clinical.	[9,89,90,91,92,93,94]
BRL-26830	β-3-adrenergic receptor	BRL-26830 causes a dose-dependent body weight loss in obese rats and mice without having this effect in lean counterparts. It increases energy expenditure with no effects on caloric intake, thereby preserving lean mass but reducing overweight.	Pre-clinical.	[95,96,97]
L-796568	β-3-adrenergic receptor	L-796568 shows high potency and specificity characteristics that could be interesting for the development of new analogs for the treatment of obesity; however, the studies conducted 20 years ago were not conclusive.	Pre-clinical.	[96,98,99]
Celastrol	Leptin	Celastrol is a leptin sensitizer that induces adipogenesis inhibition, a metabolic increase in energy expenditure, and mitochondrial gene expression in mice fed a high-fat diet.	Pre-clinical.	[100,101,102,103]
Sarcolipin	SERCA uncoupling	Sarcolipin uncouples SERCA ATP hydrolysis from Ca^2+^ transport, thereby inducing muscle thermogenesis.	Pre-clinical.	[104,105,106]
Artepillin C	PPAR-γ is a ligand-activated transcription factor	Artepillin C (ArtC) promotes thermogenesis in vivo and acts as a peroxisome proliferator-activated receptor γ (PPAR-γ) agonist.	Pre-clinical.	[107,108]

## Data Availability

No new data were created or analyzed in this study. Data sharing is not applicable to this article.
